# A Synchronous Pancreatic Metastasis from Renal Clear Cell Carcinoma, with Unusual CT Characteristics, Completely Regressed after Therapy with Sunitinib

**DOI:** 10.1155/2014/473431

**Published:** 2014-03-04

**Authors:** Salvatore Lauro, Elisa Concetta Onesti, Riccardo Righini, Francesco Carbonetti, Antonio Cremona, Paolo Marchetti

**Affiliations:** ^1^Department of Oncology, Sant'Andrea Hospital, Faculty of Medicine and Psychology, Sapienza University of Rome, Via di Grottarossa 1035, 00189 Rome, Italy; ^2^Department of Radiology, Sant'Andrea Hospital, Faculty of Medicine and Psychology, Sapienza University of Rome, Via di Grottarossa 1035, 00189 Rome, Italy

## Abstract

We present a case report of a 75-years-old woman affected by renal clear cell carcinoma with a synchronous pancreatic metastasis and a metachronous lung metastasis. This case has two peculiarities. First the pancreatic metastasis was treated just with medical therapy, that is, Sunitinib, instead of the surgical therapy that is mostly considered. Secondly, the pancreatic lesion showed different characteristics on the computed tomography scan compared to the usual pancreatic metastases from renal clear cell carcinoma. The pancreatic metastasis totally regressed after medical treatment and nowadays, four years after the diagnosis, the patient is disease-free.

## 1. Introduction

The presence of pancreatic metastases from solid tumours is a rare event, with an incidence rate varying from 1.6% to 11% in autopsy series of patients affected by advanced neoplasm [1]. The neoplasms that could give these types of metastases are melanoma, lung tumours, colic neoplasms, breast cancer, and with a higher rate of frequency the kidney tumours [2]. In many cases described in the literature pancreatic metastases due to kidney carcinoma usually manifest many years after the diagnosis of the primitive tumour [3–6]. Treatment usually is surgery, with satisfying results in terms of overall survival and disease-free survival [2–4, 7].

## 2. Case Presentation

**Figure 1:**
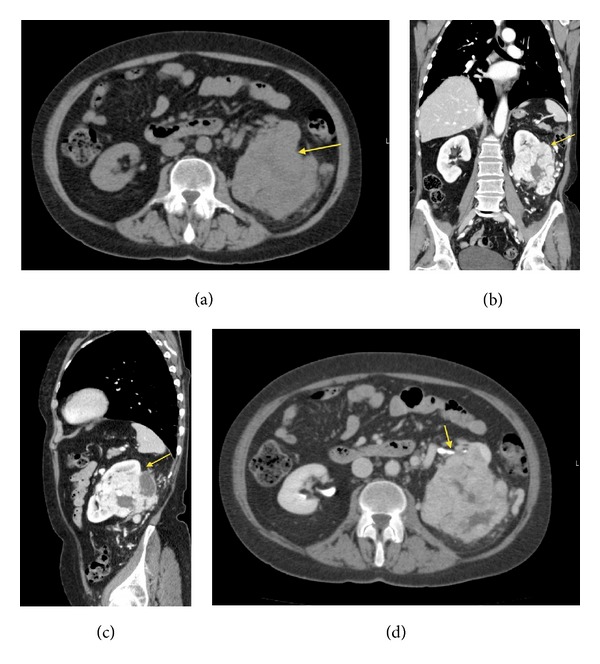
(a) and (b) show the presence of the renal lesion with multilobulated shapes, in the left kidney, 90 mm (LD) × 85 mm (TD) × 96 mm (APD) in size. (c) and (d) show the extension and the location of the renal lesion, mass infiltrates the renal pelvis and the mid calyx (c), and proximal ureter was encased but not infiltrated (d). Arrows indicate the lesion. (a) Axial CT scan, precontrast phase. (b) CECT, multiplanar reconstruction (MPR) Coronal Plane. (c) CECT-MPR: Sagittal Plane. (d) Axial-CECT, urographic phase.

**Figure 2:**
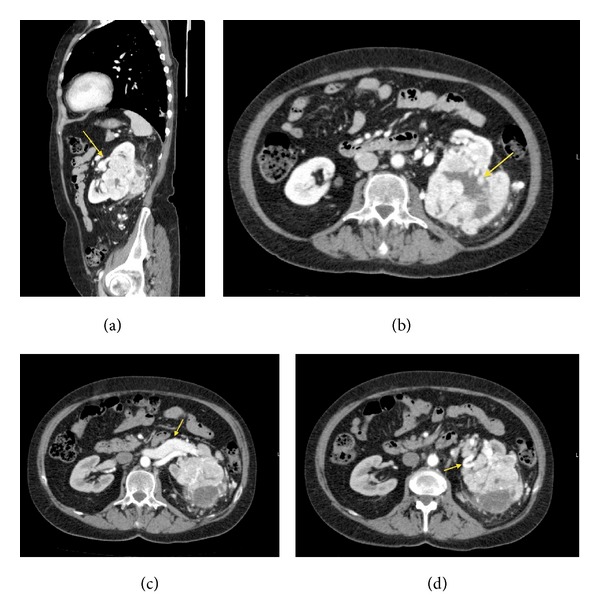
Images show the contrastographic behavior of the renal lesion and its relations with the surrounding anatomical structures. The lesion showed an inhomogeneous enhancement after contrast medium administration due to the presence of solid parts (a), and many colliquative-necrotic areas and intralesional calcifications (b) were also seen. An enlargement of the ipsilateral splenic vein, 9 mm sized, (c) and many intralesional collateral vessels (d) were seen. (a) CECT-MPR-Sagittal Plane. (b) CECT-Arterial Phase-Axial. (c) CECT-Venous phase-Axial. (d) CECT-Arterial phase-Axial.

**Figure 3:**
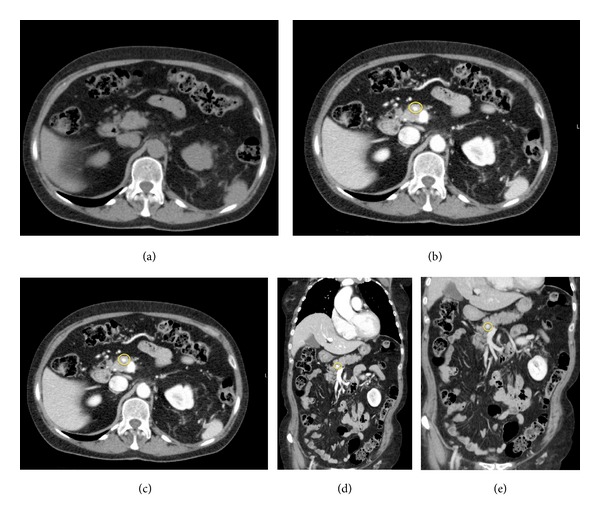
Images show the presence of a round-lesion at the pancreatic istm; the lesion hypodense in the precontrast study (a), appeared hypervascularized in all the three phases of the exam ((b), (c), (d), and (e)). Circles indicate the lesion. (a) Precontrast-Axial. (b) CECT-Arterial Phase-Axial. (c) CECT-Venous Phase-Axial. (d) CECT-Venous Phase. Coronal Plane. (e) CECT-Late Phase-Coronal Plane.

The patient, a 75-year-old female, had a positive oncological anamnesis: her brother was affected by rectal neoplasm and her three aunts were affected by breast cancer, bladder cancer, and rectal cancer, respectively. The patient was under medical treatment for hypertension and due to the onset of general malaise, anemia, and asthenia, she performed a total body contrast enhanced computed tomography (CECT) scan. CECT total body scan was performed with a triphasic study of the abdomen and with a urographic phase. The scans showed the presence of an expansive solid growing mass with multilobulated shapes on the left kidney. The lesion was 90 mm (Longitudinal Diameter-LD) × 85 mm (Transversal Diameter-TD) × 96 mm (Anteroposterior Diameter-APD) sized (Figures 1(a), and 1(b)). The mass infiltrated the renal pelvis and the mid calyx (Figure 1(c)); proximal ureter was encased but not infiltrated (Figure 1(d)). The mass showed an inhomogeneous enhancement after contrast medium administration due to the presence of solid parts; many colliquative-necrtotic areas and intralesional calcifications were also found (Figures 2(a) and 2(b)). An enlargement of the ipsilateral splenic vein, 9 mm sized (Figure 2(c)), and many intralesional collateral vessels were also observed (Figure 2(d)). No radiological signs of thrombosis of the main arteries or veins were detected. At the istm of the pancreas a nodular round-shaped lesion, 6 mm in size, was observed. The lesion, isodense in the precontrast scans (Figure 3(a)), after contrast medium administration, appeared hypervascularized in all the three phases of the study with a rich vascular supply (Figures 3(b), 3(c), 3(d), and 3(e)) and was referable, due to its contrastographic behavior, to a local metastasis or to a primitive neuroendocrine tumor. The pancreatic lesion showed on CECT scan unusual characteristics for a metastasis from renal clear cell carcinoma and, before considering surgical excision of the pancreatic mass, we decided that biopsy of the pancreatic lesion was needed in order to assess its nature. So the patient underwent a left nephrectomy with surrenalectomy and at the same time biopsy of the pancreatic lesion was performed. At the histological examination the renal mass resulted to be a clear cell carcinoma and the pancreatic lesion was found to be a metastasis of clear cell renal carcinoma.

**Figure 4:**
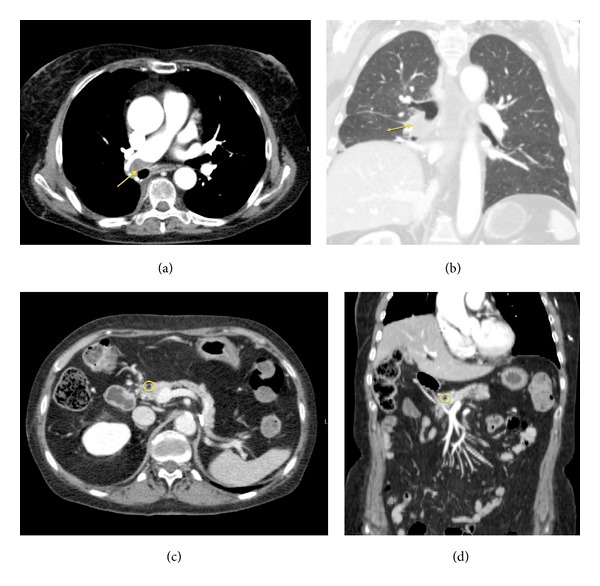
Images show the presence of the nodular lesion in the posterior basal segment of the inferior right lobe ((a) and (b)) and the pancreatic lesion after the treatment appeared hypodense due to necrotic-colliquative phenomena ((c) and (d)). (a) Axial CECT-Arterial Phase. (b) MPR-Coronal Plane-Lung Window. (c) CECT-Axial Plane. (d) CECT-MPR-Coronal Plane.

Despite the guidelines indicated a local excision of the pancreatic lesion, we decided to start a systemic treatment, considering that the patient was not able to perform a second surgery because she had just undergone a major surgical intervention and she was not in good clinical condition at that time [8]. So, the patient started medical treatment with Sunitinib, 50 mg/day for two cycles. Due to the onset of grade III skin toxicity and gastrointestinal toxicity, the dose of Sunitinib was reduced to 37.5 mg/day for other six cycles [9]. After three months of treatment another CECT total body scan was performed. CECT scan showed in the posterior-basal segment of the inferior right lobe, close to the pleural wall, a solid nodular lesion, 10 mm (LD) × 8 mm (TD) × 8 mm (APD) sized (Figures 4(a) and 4(b)). The hypervascularized pancreatic lesion was not more observed; a hypodense round-shaped lesion in the same region was observed (Figures 4(c) and 4(d)) which was a colliquative-necrotic outcome of the therapy. Considering that the lung lesion appeared during treatment with Sunitinib we thought that the best therapeutic choice was to remove surgically the lung lesion, mainly for two reasons. The first reason was that the patient was in good general clinical condition with adequate lung function and the lesion was surgically resectable. So, according to guidelines, surgery was indicated [8]. The second reason was that the lung lesion appeared during treatment with Sunitinib, while the pancreatic metastases disappeared, so there was a possibility that the lung lesion was a second malignancy. In our case we decide to surgically remove the lung lesion in order to cure the patient and also because a histological characterization of the lung lesion was needed in order to differentiate the lung lesion from a second malignancy. Therefore, the patient performed surgical removal of the lung lesion. Histological examination deposed for metastasis from clear cell renal carcinoma.

**Figure 5:**
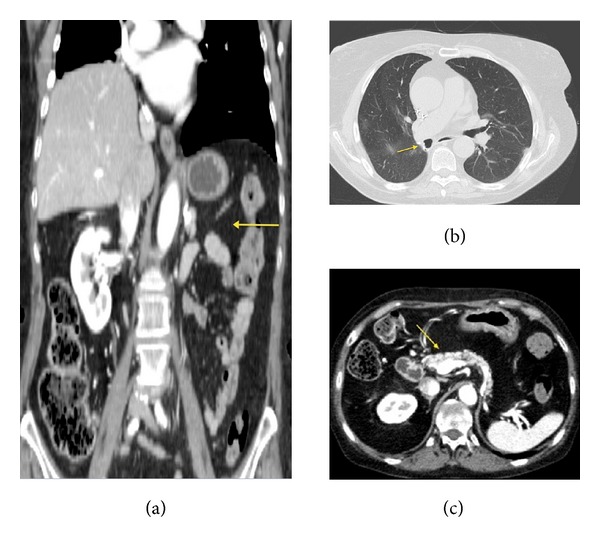
Images show the absence of local relapses after the surgery in the left kidney (a) and in the lung (b); the hypodense pancreatic lesion was not more detectable at the pancreatic istm and no other pancreatic lesions were seen (c). (a) CECT-MPR-Coronal Plane. (b) CECT-Axial. Lung Window. (c) CECT-Axial. Arterial Phase.

After the surgery the patient performed two more cycles of Sunitinib, stopped due to poor tolerance, a total of eight cycles were administrated. The following CECT exams showed the outcomes, such as metallic clips, of the left nephrectomy and surrenalectomy (Figure 5(a)), and of the resection of the lung metastasis (Figure 5(b)); no local relapses were seen in both the organs. The previous hypodense lesion seen at the istm of the pancreas totally regressed and no other pancreatic lesions were observed (Figure 5(c)). Nowadays the patient is under oncological follow-up; she is in a good state of health, and she is disease-free for 47 months from the diagnosis of the tumour and for 20 months from the end of the treatment with Sunitinib.

## 3. Discussion

The presence of pancreatic metastases from solid tumours is a rare event, with an incidence rate varying from 1.6% to 11% in autopsy series of patients affected by advanced neoplasms [1]. The neoplasms that could give this type of metastases are melanoma, lung tumours, colic neoplasms, breast cancer, and with a higher rate of frequency kidney tumours [2]. Metastasization to the pancreas is most likely due to haematogenous spreading and the site of the tumour, right or left kidney, is an independent prognostic factor of metastasization. This occurs, as described in the analysis of Sellner et al., for the high affinity of the renal cancer cells for the parenchyma of the pancreas [10]. In a review of the literature performed by Sellner et al., diagnosis in only 35% of the cases was made in asymptomatic patients. In the remaining 65% of cases, the patients had various symptoms including the following: abdominal pain (20%), gastrointestinal bleeding due to infiltration of the duodenum (20%), obstructive jaundice (9%), weight loss (9%), pancreatitis, and diabetes (3%) [10]. In many cases the metastases were metachronous with appearance 2–18 years after the diagnosis of the primary tumour [11]. The treatment of the metastases is usually surgical, with good results in terms of overall survival (about 43 months) and disease-free survival (23.6 months), while the 5-year survival after surgery is between 43% and 88% [2, 4]. Usually metastases from clear cell renal carcinoma on CT scan are isoattenuating in the unenhanced phase, as observed in our case. After contrast medium administration pancreatic metastases from renal clear cell carcinoma usually closely resemble the appearance of the primary carcinoma, showing on CECT scan irregular and not well-rounded shapes, a nonhomogenous and irregular contrast enhancement, and a central hypoenhancing necrotic portion [12–14]. Despite these general features, in our case the pancreatic metastasis on CECT scan showed well-rounded shapes and a homogenous and uniform contrast enhancement without any hypoenhnacing necrotic portion. According to the literature and to our daily radiological experience small hypervascular lesions, with rounded shapes and with a rich and uniform contrast enhancement, as observed in our case, rarely could be referred to metastases from renal clear cell carcinoma. Most often small pancreatic lesions, with rounded shapes and with a uniform contrast enhancement on CECT scan, could be referred to neuroendocrine pancreatic tumours, primitive or metastatic to the pancreas [12–14]. For these reasons the pancreatic lesion observed in our case presented, on CECT scan, unusual characteristics for a metastasis from renal clear cell carcinoma and this is why the biopsy of the lesion was performed.

In our case the diagnosis of the pancreatic metastasis is synchronous to the primary tumour, unlike the majority of the cases reported in the literature, where the metastases appeared many years after the diagnosis of the primary tumour. In our case the symptomatology was very mild and nonspecific, not clearly indicative of the presence of a pancreatic lesion. Although the guidelines indicate the need for a complete local excision, in our case the patient, who had just performed a major abdominal surgery with nephrectomy, was not in suitable clinical conditions to undergo a second surgical procedure [8]. Thus, the type of treatment we have chosen was pharmacological, using Sunitinib an inhibitor of the tyrosine kinase receptor. Regarding the pulmonary mass, because it appeared during the treatment with Sunitinib, we had thought it was probably a selection of a clone of resistant cells or a second malignancy, so, considering that the patient at that time was in a good clinical condition to perform another surgery, the best therapeutic choice was the surgical resection in order to make a correct differential diagnosis and to obtain a complete eradication of the lung lesion. The management of lung metastases while on Sunitinib treatment must be assessed individually. The target is to reach the complete eradication of the disease with a multimodal approach. Unfortunately, currently there are no randomized trials designed to gain information about the correct timing of treatment strategy [15]. The data reported in the literature show that metastasectomy after initial systemic therapy gave partial or complete response in a majority of patients. In these patients the median survival was 4.7 years [16]. In patients with a single resected pulmonary metastasis a 5 year-overall survival of 50% has been reported [17]. Moreover, it must be considered that there is a difference if the lung metastasis is already present before starting treatment with Sunitinib or if it appears during therapy. In the second case a II line pharmacological treatment is indicated, if metastasectomy is not possible [18]. Regarding the choice to continue or discontinue the systemic treatment after achieving a complete response, in the literature there are some studies on few patients, so the results are not yet conclusive [19, 20]. It has been suggested that, after complete response, there may still be residual cancer cells [19]. Some articles published in literature affirm that the discontinuation of chemotherapy may allow the cells to proliferate and lead to disease recurrence, whereas continuation of chemotherapy maintains therapeutic pressure on the residual cancer cells, preventing disease recurrence [20]. Based on these data we continued treatment after achieving a complete response but, due to poor tolerance of the patient, we had to stop the treatment.

However our patient, 47 months after the diagnosis and 20 months after the end of the therapy, is in good condition and disease-free. It is important to remember that patients with pancreatic metastases from renal cancer could be symptomatic but also asymptomatic. Considering that renal clear carcinoma could give metastases even after many years the remission of the disease, a long time follow-up with periodic radiological exams should be performed in patients who had a renal clear carcinoma. Even if the treatment for pancreatic metastasis from renal clear carcinoma is surgical, also the medical therapy for the metastatic lesions could be considered and each case must be assessed individually.
